# Modelling intra-muscular contraction dynamics using in silico to in vivo domain translation

**DOI:** 10.1186/s12938-022-01016-4

**Published:** 2022-07-08

**Authors:** Hazrat Ali, Johannes Umander, Robin Rohlén, Oliver Röhrle, Christer Grönlund

**Affiliations:** 1 Department of Electrical and Computer Engineering, COMSATS University Islamabad, Abbottabad Campus, Abbottabad, Pakistan; 2 Department of Radiation Sciences, Umeå University, Umeå, Sweden; 3 Stuttgart Center for Simulation Technology (SC SimTech), University of Stuttgart, Stuttgart, Germany; 4 Institute for Modelling and Simulation of Biomechanical Systems, Chair for Computational Biophysics and Biorobotics, University of Stuttgart, Stuttgart, Germany

**Keywords:** Domain adaptation, Noise adaptation, Generative adversarial network, Neural networks, Skeletal muscle, Ultrasound, Plane wave, High frame rate imaging, Fascia, Simulation model

## Abstract

**Background:**

Advances in sports medicine, rehabilitation applications and diagnostics of neuromuscular disorders are based on the analysis of skeletal muscle contractions. Recently, medical imaging techniques have transformed the study of muscle contractions, by allowing identification of individual motor units’ activity, within the whole studied muscle. However, appropriate image-based simulation models, which would assist the continued development of these new imaging methods are missing. This is mainly due to a lack of models that describe the complex interaction between tissues within a muscle and its surroundings, e.g., muscle fibres, fascia, vasculature, bone, skin, and subcutaneous fat. Herein, we propose a new approach to overcome this limitation.

**Methods:**

In this work, we propose to use deep learning to model the authentic intra-muscular skeletal muscle contraction pattern using domain-to-domain translation between in silico (simulated) and in vivo (experimental) image sequences of skeletal muscle contraction dynamics. For this purpose, the 3D cycle generative adversarial network (cycleGAN) models were evaluated on several hyperparameter settings and modifications. The results show that there were large differences between the spatial features of in silico and in vivo data, and that a model could be trained to generate authentic spatio-temporal features similar to those obtained from in vivo experimental data. In addition, we used difference maps between input and output of the trained model generator to study the translated characteristics of in vivo data.

**Results:**

This work provides a model to generate authentic intra-muscular skeletal muscle contraction dynamics that could be used to gain further and much needed physiological and pathological insights and assess and overcome limitations within the newly developed research field of neuromuscular imaging.

## Introduction

Investigation of skeletal muscle contraction is commonly conducted in many sports medicine applications, rehabilitation, and diagnosing neuromuscular disorders. State-of-the-art methods rely on investigating the smallest functional units, i.e. the motor units (MUs), and record the electrical activity of their muscle fibres (electromyography). These methods are, however, limited to a small field-of-view [1, 2]. Recently, this limitation has been addressed by introducing large-field-of-view methods for imaging individual MUs of the skeletal muscle contraction, using tissue mechanics acquired by ultrafast ultrasound imaging and magnetic resonance imaging [3–9]. While the bioelectrical methods only record the electrical activity of muscle fibres, the mechanical activity is more complex due to the composition of the muscle tissue.

The skeletal muscle tissue comprises muscle fibres embedded in a multilevel complex web of connective tissues. In addition, skeletal muscle tissue includes vascular tissue, skin and subcutaneous fat, potential fibrosis, fat tissues within the muscle, bone, etc. However, due to large variations in the amount of these components, there is a lack of knowledge on the effect of mechanical coupling on its physiology [10–13]. To model the full complexity and heterogeneity of skeletal muscle tissue, a 3D continuum-mechanical approach is essential. Such models are rare. Moreover, most of these models follow a phenomenological modelling approach to describe a skeletal muscle’s mechanical behaviour, e.g., [14–17]. Furthermore, these studies investigate muscles in isolation, i.e. without taking into account their mechanical state at rest (e.g., pre-stretch), adjacent tissues, or heterogeneous material descriptions. They rely on constitutive model parameters obtained by fitting phenomenological constitutive laws to experiments (often conducted in animals). Some exceptions exist. For example, they consider the inclusion of the electro-physiological behaviour of skeletal muscle fibres [18], or develop micro-mechanical models investigating the mechanical behaviour of a few muscle fibres or fascicles [19, 20]. All these models yet appeal to bulk properties. The inclusion of heterogeneous material distributions is feasible but typically not considered. To realistically model variations in collagen distributions and dispersion within the extracellular space and deduce from that the overall mechanical behaviour of the muscle tissue, a new class of skeletal muscle models based on novel homogenization techniques have been proposed [21, 22]. These, however, currently only consider the passive mechanical properties. Fully dynamic models that take into account the microstructure are currently missing, but are needed to improve image-based model assessment of muscle contractions. Further, from a computational point of view, the requirements on spatial and temporal resolution (0.1mm and kHz range) to simulate skeletal muscle contraction make the use of models described above nearly infeasible. A simulation model to generate data with authentic features would provide a valuable tool to advance the research field on imaging MUs in skeletal muscle contraction.

Deep learning generative models have recently been proposed as a tool to simulate medical images [23–25] and, in specific, ultrasound images [26–28] with authentic features. However, these models generate 2-D images, whereas skeletal muscle contractions are characterized by repeated contractions (twitches) of the activated fibres [29]. As a consequence, a model including spatio-temporal features is required to simulate authentic skeletal muscle contractions (i.e. image sequences, e.g., 2-D + time), but studies on such application are lacking. Domain-to-domain translation is a particular branch of deep learning generative models, which allows to transfer data from one domain to another with different feature distributions while retaining content [30–36]. Thus, such models may offer a solution to generate authentic experimental domain (in vivo) spatio-temporal data from simulated domain (in silico) spatio-temporal data.^1^

In this work, we aim to model intra-muscular skeletal muscle tissue contractions, by generation of authentic image sequences using in silico (simulated) to in vivo (experimental) domain translation. We modified and trained a 3D cycle generative adversarial network (cycleGAN) model [35, 37, 38] using unpaired in silico and in vivo image sequences, and evaluated hyperparameter settings on the domain adaptation performance by quantitative comparison of spatial and temporal domain features. Our goal in this work is to learn a mapping where the underlying content is preserved while the domain-specific features of the experimental data should be transferred to the simulated content. To the best of our knowledge, there are no previous works on domain adaptation of ultrasound image sequence data (video), where spatio-temporal consistency needs to be retained when adapting to the new domain.

Our main contributions are:

1. A modified 3D cycleGAN model to generate authentic image sequences of skeletal muscle tissue contraction from in silico image sequences (while maintaining spatio-temporal consistency of the content in the in silico data),
2. A description of the spatial features of authentic in vivo and the in silico data for the first time, and a demonstration that there are large differences,
3. The use of difference maps between the input–output paired data (in silico and translated domain) to assess the learned characteristic feature representation of the model’s generator, and
4. The first study applying domain–domain translation on medical image sequences.

## Generative models in medical image synthesis

Generative adversarial networks (GANs) have gained popularity for their ability to generate authentic synthetic medical image data [23–25, 35, 37, 39]. GANs are increasingly becoming popular in the medical imaging and medical ultrasound imaging research community [23]. Recently, Lennart et al. [27] proposed SpeckleGAN for ultrasound image simulations. The architecture is a GAN model with the exhibition of speckle noise to reflect more realistic distribution. Cronin et al. [40], demonstrated the use of GANs for synthesis of musculoskeletal B-mode ultrasound images from synthetic segmented masks. The model is a traditional cycleGAN for 2D images. Hu et al. [41] have demonstrated the use of conditional GANs for simulating foetal ultrasound images. The GAN is conditioned on calibrated pixel coordinates in global physical space. For low-resolution portable ultrasound devices, Wang et al. generated super resolution with the help of GANs [42]. The model uses two generators with U-Net architecture to build a sparse skip connection U-Net. Fujioka et al. [26] used deep convolutional GAN to generate breast ultrasound images and to express virtual interpolation images of tumours. The readers may also be interested in the survey paper on the applications of GANs for synthesis of radiology images [43].

Most of the GAN-based studies applied to ultrasound imaging (and generally, in medical imaging) focus on the generation of 2D synthesized data. These studies are limited to image-to-image translation approaches with no inclusion of temporal features. In comparison to these works, the present work addresses the task of US image sequence generation—a video-to-video translation.

For medical image volume data, Abramian et al. [44] demonstrated synthesis of functional magnetic resonance imaging (fMRI) volumes from T1-weighted volumes using a 3D cycleGAN architecture. Similarly, synthesis of 3D volumes of MRI was described by Zhang et al. [35] where they used a shape-consistency loss for the generators of the GAN to achieve a synthesis with authentic features. These two approaches can also be regarded as volume-to-volume translation application of GANs in medical imaging.

There are a few studies on video-to-video translation for video of natural scenes. For example, Bansal et al. [33] presented the Recycle-GAN model to synthesize future frames. By using a temporal predictor, a cycle-consistency was explored across both domains as well as along time. In addition, Chen et al. [34] applied a motion-guided cycleGAN to explore both structure appearance and temporal continuity for video-to-video translation tasks such as flower-to-flower translation. However, to the best of our knowledge there are no proposed models for medical and ultrasound image sequence (video-to-video) translation, and in particular not for skeletal muscle contraction dynamics.

**Fig. 1:**
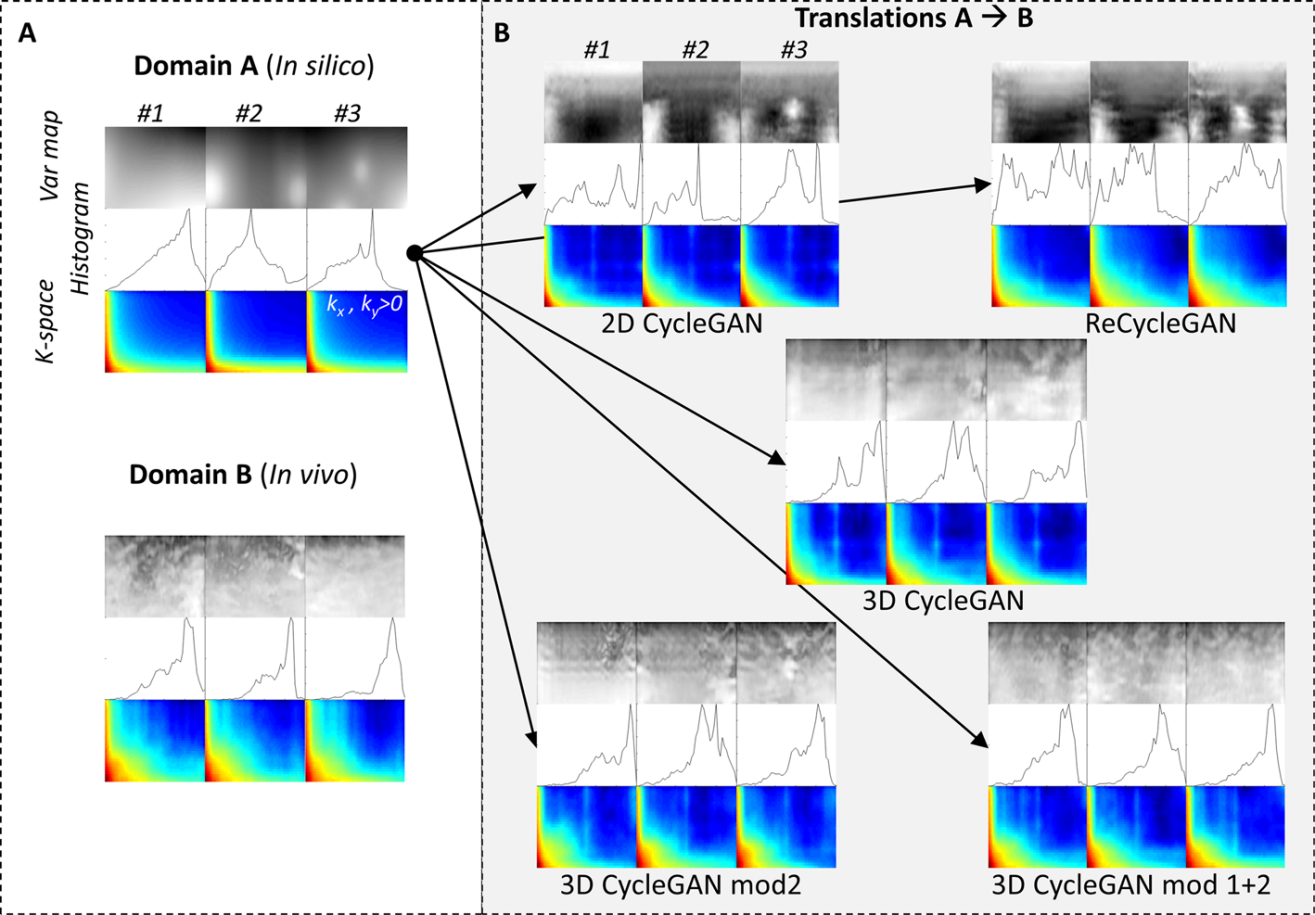
**A** Examples of spatial variance maps (var map) and their spatial features represented in histogram and K-space (positive frequencies only), from domain A and B data, respectively. **B** Examples of spatial variance maps resulting from the trained translation models 2D cycleGAN, reCycleGAN, and the 3D cycleGAN models. The translated examples are derived from 1-3 in (**A**). The image sequences are here represented by a variance map, its corresponding histogram, and K-space (2D Fourier transform, positive frequencies)

**Table 1:** Performance evaluation of domain–domain translation models

Direction	Translation model	Frames	Spatial features			Temporal features	Translated content consistency	
			DBhat $\downarrow$	Corr $\uparrow$	SSI $\uparrow$	Corr Psd $\uparrow$	xCorr $\downarrow$	Lag, ms $\downarrow$
Sim to Exp	No translation	–	0.33 (0.11)	0.54 (0.26)	0.32 (0.18)	0.96 (0.01)	–	–
-”-	2D cycleGAN	1	0.43 (0.10)	0.32 (0.19)	0.49 (0.12)	0.90 (0.02)	0.61 (0.09)	− 2.0 (64.6)
-”-	Recycle GAN	3	0.39 (0.11)	0.39 (0.20)	0.65 (0.14)	0.96 (0.01)	0.51 (0.07)	− 1.9 (70.8)
-”-	3D cycleGAN default	32	0.25 (0.09)	0.67 (0.21)	0.49 (0.08)	0.90 (0.02)	0.89 (0.04)	1.3 (2.4)
-”-	3D cycleGAN (Stride=1)	32	0.27 (0.12)	0.65 (0.25)	0.63 (0.09)	0.97 (0.01)	0.93 (0.03)	− 1.7 (2.2)
-”-	3D cycleGAN (Stride 1 + Noise)	32	0.25 (0.10)	0.69 (0.23)	0.65 (0.10)	0.97 (0.01)	0.93 (0.03)	1.4 (2.2)
Exp to Exp	No translation	–	0.26 (0.11)	0.66 (0.25)	0.74 (0.12)	0.98 (0.01)	–	–

Spatial and temporal features were computed as cross-comparisons between translated and experimental sequences

*D Bhat* Spatial features were compared using Bhattacharyya distance, *Corr* histogram correlation,*SSI* structural similarity index of K-spaces, and *Corr Psd* temporal features was compared using correlation of power spectral densities . *xCorr* The consistency of the signal content in the translations was assessed using cross-correlation and *Lag* the corresponding time lag

**Fig. 2:**
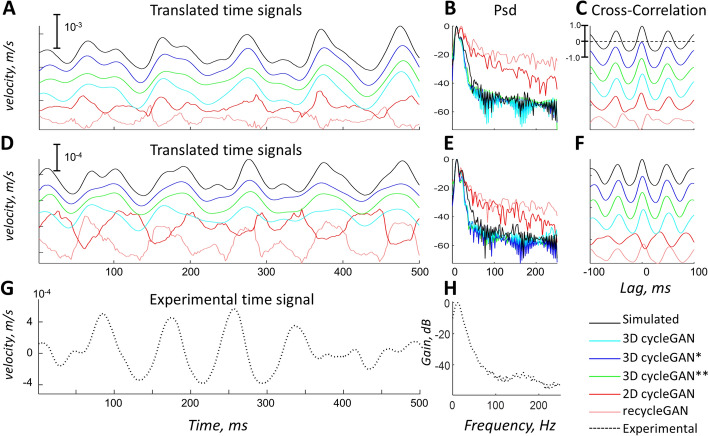
Examples of two sets of translated time signals (**A** and **D**), their corresponding power spectral densities (Psd) (**B** and **E**), and cross-correlation with the input simulated signal (**C** and **F**). The original signal as well as that the outputs from the different translations are shown in different colours in time traces. Content similarity was computed as the maximal cross-correlation between the translated signals and the original simulated signal. It can be seen that the translations of the 3D cycle GAN models were similar to the simulated signals, whereas the translations from the 2D GAN and the recycle-GAN models were not and came with large amount of noise. **G** Shows an example of an experimental time signal, and **H** its corresponding psd. It should be stressed that the oscillatory pattern is similar in simulated and experimental signals. $$*$$ *= modification 2 (stride 1),* $$**$$ = *modification 1*$$+$$*2 (stride 1 and noise injection)*

## Results

### Comparison between domain A and domain B features

The spatial features of the simulated (domain ${\mathbf {A}}$) and experimental (domain ${\mathbf {B}}$) data were different (see Fig. 5 later in the text), both at the level of individual frames of an image sequence, as well as the computed variation maps. In particular, in the simulated data individual MUs could be seen as regions with oscillating intensities (black to white). Regions with oscillating intensities could also be observed in the experimental data, but the spatial pattern was more complex. This dissimilarity was also verified in the quantitative assessment with high DBhat (0.33), low Corr (0.54), and low SSI values (0.32) (Table 1, first row, No-translation case). In contrast, the temporal content was similar, as shown by a high correlation of the power spectral content (Corr Psd 0.96) between simulated and experimental data, which was also on the same order as when comparing experimental sequences with other experimental sequences (0.98) (Table 1, first and last rows). Examples of time signals and their Psd from both domains can be seen in Fig. 2.

### Evaluation of translation performance

Figure 1 shows three examples of the original domain ${\mathbf {A}}$ and ${\mathbf {B}}$ data and their corresponding five different translations. Table 1 shows the quantitative results of comparing spatial, temporal, and spatio-temporal features between the translated and the experimental data.

#### Spatial features

The 2D cycleGAN and recycleGAN models had the lowest spatial feature similarity compared to the experimental data as indicated by the high DBhat and low Corr values. The 3D cycleGAN models had similar performance in generating authentic spatial features, and was similar to when comparing experimental data with itself (DBhat 0.25–0.27 vs 0.26; Corr 0.65-0.69 vs 0.66). The SSI was highest for the 3D cycleGAN models with stride 1 (0.63 and 0.65) but not as high as when comparing experimental data with itself (0.74). In Fig. 1, it can be seen that the default 3D cycleGAN model presented variation maps with a smoothed spatial features. The 3D cycleGAN model with stride 1 resulted in a relatively periodic spatial pattern, and the 3D cycleGAN model with stride 1 and noise injection presented visual features similar to those of the experimental data. Taking the spatial features together, the stochastic noise injection model produced the most authentic spatial features based on having the highest Corr (0.69), SSIM as high as the best of all models (0.65) and with DBhat as low as the best of all models (0.25).

#### Temporal features and translated content consistency

Figure 2 shows examples of the typical oscillatory content of the signals and indicates large differences between the translations. In general, the Corr Psd was similar among the translation models (0.90-0.97) and was comparable to that of the experimental data (Corr Psd 0.98), but the 2D cycleGAN model had the lowest similarity (Table 1). The 2D cycleGAN model and ReCycleGAN models had poor performance in translating the content (low xCorr values and large variation in the lag). In contrast, the 3D cycleGAN models translated the content to a large extent (xCorr 0.89-0.93, lag close to zero and low standard variation).

Taken together, the 3D cycleGAN models retained spatio-temporal consistency, and produced spatial and temporal features similar to those of comparing experimental data with itself. The model with stochastic noise injection produced the most authentic spatial features while simultaneously having the top rank performance on temporal feature authenticity Corr Psd (0.97) and top rank performance on preserving the temporal content xCorr (0.93). Thus, it had the most authentic spatio-temporal features.

**Fig. 3:**
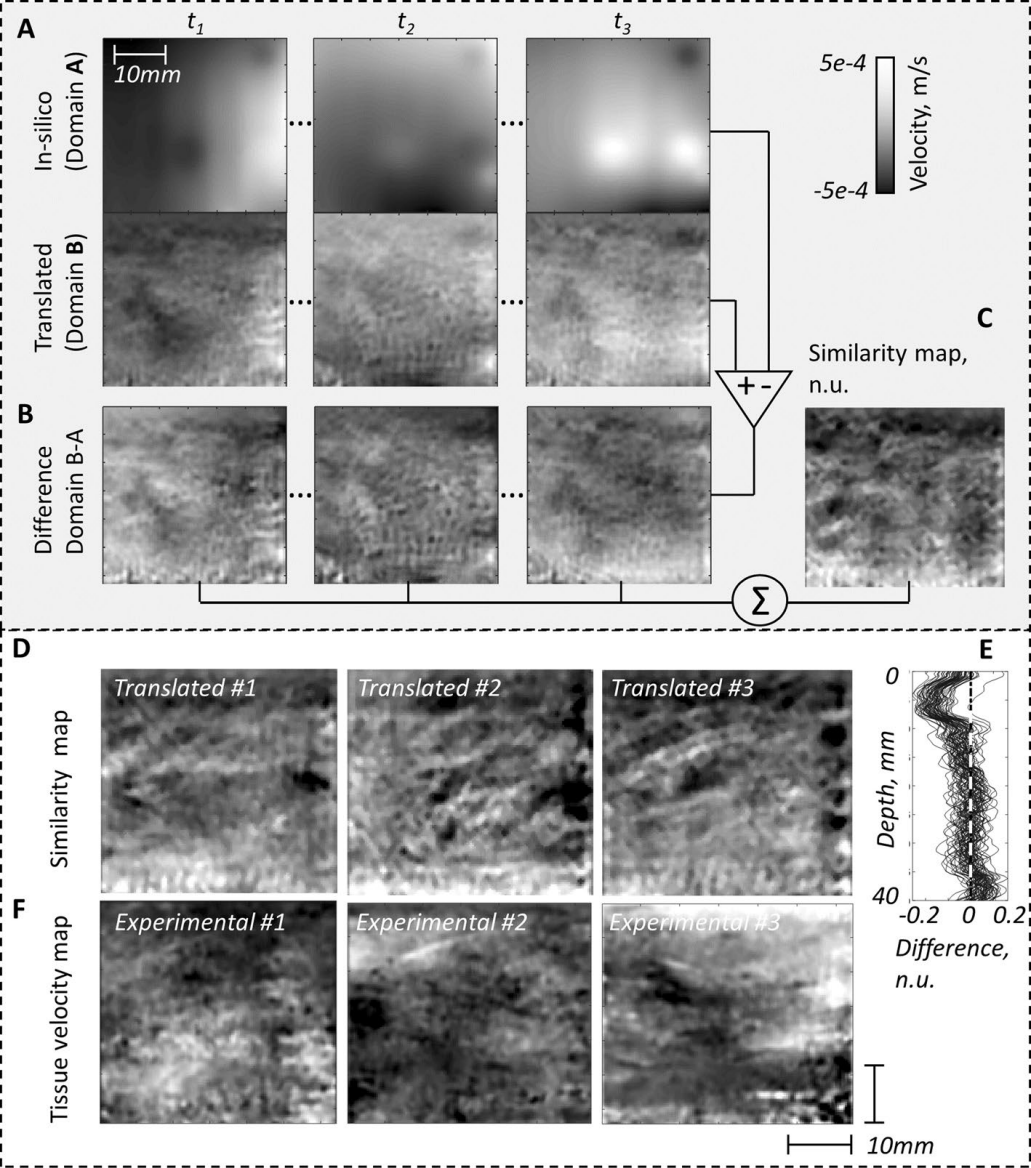
Exploration of the learned mapping of the generator model. **A** Examples of simulated (in silico), translated tissue velocity images, **B** their corresponding differences at three different time frames of an image sequence, and **C** is the similarity map computed as the sum of difference maps of the whole image sequence. **D** Similarity maps for three translated examples. **E** Similarity as a function of depth for all 64 translated examples. The similarity was low in superficial subcutaneous region. **F** Examples of experimental tissue velocity frames. It can be seen by visual inspection that the texture pattern of the similarity maps (**D**) shared similar features with the texture pattern of the experimental velocity maps (**F**)

### Assessment of the learned mapping of the generator

Figure 3A and B shows examples of simulated and translated image frame pairs of a sequence for the 3D cycleGAN model (stride 1 and noise injection), and their corresponding difference maps. The resulting sum of the difference maps of a sequence was denoted similarity map (Fig. 3C) and this was used to assess the spatial features that the generator had learned to translate to the simulated data. The translated spatial features, as assessed by the similarity maps, were similar but not identical in the translated sequences (Fig. 3D), and it was similar to the spatial features of the experimental data frames (Fig. 3F). In the similarity maps, the difference was approximately 0 (large similarity) but with a granular pattern of holes with regional differences (1–2 mm diameters). The experimental images presented a similar spatial texture pattern of variations in tissue velocity. The translated data had lower velocities in the subcutaneous (superficial) region (Fig. 3E).

## Discussion

In this work, we evaluated different domain–domain translation models to generate authentic image sequences of intra-muscular contraction dynamics. The main findings of this work are: (1) there were large differences between the spatial features of the simulated and experimental domains before translation; (2) a domain–domain translation model can be trained to generate authentic data and that a 3D but not 2D model is required to obtain spatio-temporal consistency, and (3) mapping of differences between input and a translated sequence showed a spatial texture which was similar to that of experimental data. This work is the first work on video-to-video domain translation task on medical image sequences, and in particular ultrasound image sequences.

### Differences between simulated and experimental domains

First, there was a large difference in the spatial features of the two domains (Fig. 1 and Table 1). This was expected and demonstrate that the experimental in vivo data comprise complex mechanical interactions between the fascia, muscle fibres, vasculature, skin and subcutaneous tissues, etc., that are not represented in the simulation domain. These results also highlight the impact and magnitude of these complex interactions on the micro-mechanical dynamics within the muscle. This has, to the best of our knowledge, not been shown before, and these findings also motivate the importance of the present work.

### Performance of domain translation models

All models resulted in adaptation towards spatial-temporal features of the experimental domain, but the 3D models performed the best. The 2D and few-frame models (2D cycleGAN and recycleGAN) resulted in similar spatial features as those of experimental data, however, temporal content was poorly translated. This was expected since the skeletal muscle contractions comprise activations of the MUs that produce an oscillating dynamical sources. Thus, in order to retain spatio-temporal consistency, a spatio-temporal model is required (3D, i.e. 2D+time).

In general, the translated sequences of the 3D cycleGAN models had a high similarity in spatial features compared with experimental data, and with a retained temporal consistency. The 3D model with deterministic generator as well as stochastic generator gave similar performance in the evaluated metrics, however visual inspection showed that checker-boarding pattern was reduced in the stochastic version. The checkerboard pattern was seen in the output of several models as repeating patterns in the variance maps as well as lines in the K-space representations (Fig. 1), particularly for the default 2D and 3D cycleGAN models. The pattern was mainly present in areas with low activity or with no active MUs. These areas correspond to low entropy and thus, these will not be optimal as priors to the pseudorandom number generators in the deterministic cycleGAN models.

One of the 3D cycleGAN model modifications was to use a stride value 1 in the third dimension corresponding to the temporal information. A value higher than one would down-sample the data and lose temporal information about the muscle contraction. While our choice of stride value was empirical, this value has also been used for 3D CNNs of other cycleGANs models for medical imaging, for example, generating functional magnetic resonance imaging (fMRI) volumes [44]

The stochastic 3D cycleGAN model showed less repeated spatial patterns and provided the best spatial feature authenticity compared to the other models (Fig. 1 and Table 1). Thus, the introduction of stochastic noise to the latent space helped achieve a generalized behaviour of the generator. This finding is in line with results obtained in the computer vision domain, for example, in face synthesis [45], where noise input has been used to improve the stochastic variation in generated images. From an anatomical and physiological perspective, the skeletal muscles of different subjects will have both common and diverse geometries and constituents [13] that should cause an inter-subject variation in some features of the texture pattern of the intra-muscular dynamics. Therefore, another advantage of the stochastic model is that it provides a new spatial feature pattern for each generation, which might be preferred from a simulation point of view.

Taken together, the 3D cycleGAN model with stochastic noise injection performed the best and is best suited for the purpose of translating authentic experimental data features.

### Assessment of the learned mapping of the generator

The difference maps showed a general high similarity between the images, but with a mesh of circular regions scattered in the images (1–2 mm diameter). Such texture pattern could be caused by the skeletal muscle fascia structures. In particular, the primary perimysium surrounds some hundreds of muscle fibres encapsulated by a layer of connective tissue. The observed circular regions in this work are on the same order of size as the typical perimysium structures [46]. One function of the perimysium is believed to facilitate deformation of the muscle during contraction, due to presence of hyaluronic acid between perimysia fascicles. Neither perimysium, nor other fascial structures are present in the simulated data and therefore these observations in the translated data indicate that the difference mapping may provide an important tool to study the intra-muscular contractions and its complex interactions.

### Potential applications

The model and approach used in this work could be used for several purposes.

First, the model allows realistic modelling of the intra-muscular contraction patterns which could be used in method development and evaluation. The recent advent of imaging techniques for motor unit identification and quantification [3–9] utilize dynamics of intra-muscular contraction patterns, and the model may assist in further development of imaging methods. For example, the simulation model could be used for data augmentation and training of deep learning models for MU identification such as in Ali et al. [47] when large amounts of training data are required. The initial simplified simulation model provides the labelling of the data and the domain transfer adapts the data to authentic experimental spatio-temporal features.

Second, the proposed assessment of the learned mapping of the generator (similarity maps) may provide a way to get insights into the characteristic tissue dynamical features of other muscles or conditions. This is important because the dynamics of the interaction of, e.g., the fascia and muscle fibres in vivo is poorly understood [11, 48]. For example, as different muscles have different compositions and architecture [46], retraining the translation model on simulated data (domain A) and corresponding experimental data from a specific muscle (domain B) may allow muscle-specific modelling and assessment of its detailed tissue dynamic texture. Moreover, skeletal muscle tissues may be affected by different diseases and ageing (sarcopenia) [13]. Therefore, the influence of, e.g., age on the mechanical dynamics pattern could potentially be studied by retraining the model to translate between data of young (domain A) and old (domain B) subjects. Applying the similarity map concept might then be used to study the influence of age between data of the young subjects, and their corresponding old-translated versions.

### Limitations

In the present work, we applied cycleGAN models. There are many other models that may have provided equal or better performance, such as starGAN [49] or DiscoGAN [50]. In addition, influence of hyperparameters, such as the number of frames of the 3D cycleGAN model may have influenced the performance. However, in this work we were primarily interested in proof-of-concept. Moreover, the choice of 32 frames for the 3D models corresponded to approx 64 ms, and was limited by memory issues, but experiments with downsampling the ultrasound image sequence by a factor 2 to 256 Hz (providing an effective receptive field of 128 ms) did not change performance (data not shown). Therefore, we believe that this was not a critical parameter.

A mixed-precision modification to the model was used to combine 16-bit and 32-bit computations. However, caution is advised when training is done in mixed precision to handle the memory resources properly.

To explore the learned mapping of the generator, we applied similarity maps. Other methods should also be assessed in future studies, e.g., the concept of exploring the trained generator latent space [26].

The choice of simulation model may have influenced the results. As previously pointed out, there are many different simulation models including a variety of parameters. For example, MU territories were here modelled as circular territories, but in reality they can have different shapes [6]. In order to understand how critical the simulation model was, we also trained the 3D cycleGAN model using a simplified version of the simulation model: territories were reduced to single spatial point, and twitch signal was reduced to a single Dirac pulse (i.e. the firing pattern). In order for the gradient descent approach to work in the training of the models, the spatio-temporal derivatives need to be smooth, and therefore we first added white noise (${\mathcal {N}}(0,0.1)$) to the sequence and then applied a spatio-temporal (3D) convolution (low-pass filter). The translated data of the resulting model presented similar features as the models trained on the full simulation model (results not shown). This indicated that the precise choice of simulation model was not critical for our results.

## Conclusion

In this work, we evaluated different cycleGAN models to generate authentic image sequences of skeletal intra-muscular contraction dynamics. Prior to translation, there were large differences in spatio-temporal features of simulated and experimental domain data. Results showed that a 3D (2D+time) cycleGAN model but not a 2D cycleGAN model could be used to generate authentic tissue velocity image sequences. Taken together, the model could learn a mapping between in silico and in vivo ultrasound image sequences where the underlying content was preserved while the domain-specific features of the in vivo data were transferred to the in silico data. To the best of our knowledge, this is the first study on domain–domain translation of ultrasound image sequence data (video), where spatio-temporal consistency needs to be retained when adapting to the new domain.

## Methods

### 3D CycleGAN architecture

In this work, we propose to achieve the domain–domain translation using the concept of the cycleGAN model [37] extended to 3D, inspired by previous applications on medical image volumetric data [35, 44] and natural videos [33, 34]. The cycleGAN model [37] exploits an architecture with two GANs working in the opposite direction, i.e. transformation $A \rightarrow B$ by generator $G_{B}$ and transformation $B \rightarrow A$ by generator $G_{A}$. The cycleGAN includes a cycle-consistency loss that improves the overall quality of the generated domain data. Unlike pix2pix GAN [38] where paired data are required for training, in cycleGAN, the examples from the two domains do not have to be paired. This implies that we can provide a training set consisting of $\{a_{i}\}_{i=1}^{N} \left( a_{i} \in A \right)$ and $\{b_{j}\}_{j=1}^{M} \left( b_{j} \in B \right)$, with no one-to-one mapping required between the examples of the two domains. *This is a requirement by our application where the in silico and in vivo domain data are un-paired.* The generators $G_{B}:A \rightarrow B$ and $G_{A}: B \rightarrow A$ are functions used to create a mapping between the two domains. The two adversarial discriminators $D_{A}$ and $D_{B}$ try to determine whether a given example is from real data or generated data.

In cycleGAN there are two objective functions, one for each domain and they are typically expressed as:

1 $$\begin{aligned} {\mathcal {L}}_{GAN_{A}}&={\mathbb{E}}_{a \sim p_{\text{data}}(A)}\left[ \log  ( D_{A}(a) ) \right] \\ &\quad +{\mathbb {E}}_{b \sim p_{\text{data}} (B)}[ \log ( 1-D_{A} ( G_{A}(b) ) ] , \end{aligned}$$

2 $$\begin{aligned} {\mathcal{L}}_{GAN_{B}} &={\mathbb {E}}_{b \sim p_{\text{data} }(B)}\left[ \log \left( D_{B}(b)\right) \right] \\ &\quad+{\mathbb {E}}_{a \sim p_{\text{data} }(A)}\left[ \log \left( 1-D_{B}\left( G_{B}(a)\right) \right] \right. , \end{aligned}$$

where $a \sim p_{\text{ data } }(A)$ and $b \sim p_{\text{ data } }(B)$ denote the data distributions, respectively.

The discriminators will try to maximize these objectives by correctly classifying real and fake (generated) data while the generators try to minimize these objectives by generating data that the discriminator incorrectly classifies as real.

The adversarial losses can cause the generated data to match the data distribution of the target domain. It will, however, not constrain the optimization to produce a desired mapping between the domains where the contents of the image are preserved while only changing the domain-related part. CycleGAN attempts to produce the desired mapping by constraining the mapping functions to be cycle-consistent, i.e. real data from domain ${\mathbf {A}}$ should be able to be translated to domain ${\mathbf {B}}$ and then translated back to domain ${\mathbf {A}}$ with a minimal difference between the original data in domain ${\mathbf {A}}$ and the cycled data.

The corresponding losses ${\mathcal {L}}_{\text{ cycle } _{A}}$ and ${\mathcal {L}}_{\text{ cycle } _{B}}$ can be expressed as:

3 $$\begin{aligned} \begin{aligned} {\mathcal {L}}_{\text{ cycle } _{A}}&=\left\| A_{\text{ cycle } }-A_{\text{ real } }\right\| _{1}={\mathbb {E}}_{a \sim p_{\text{ data } }(A)}\left\| G_{A}\left( G_{B}(a)\right) -a\right\| _{1}, \\ {\mathcal {L}}_{\text{ cycle } _{B}}&=\left\| B_{\text{ cycle } }-B_{\text{ real } }\right\| _{1}={\mathbb {E}}_{b \sim p_{\text{ data } }(B)}\left\| G_{B}\left( G_{A}(b)\right) -b\right\| _{1}. \end{aligned} \end{aligned}$$

With hyperparameters $\lambda _{A}$ and $\lambda _{B}$, the total loss becomes:

4 $$\begin{aligned} {\mathcal {L}}_{\text{ total } }={\mathcal {L}}_{G A N_{A}}+{\mathcal {L}}_{G A N_{B}}+\lambda _{A} {\mathcal {L}}_{\text{ cycle } _{A}}+\lambda _{B} {\mathcal {L}}_{\text{ cycle } _{B}}. \end{aligned}$$

**Fig. 4:**
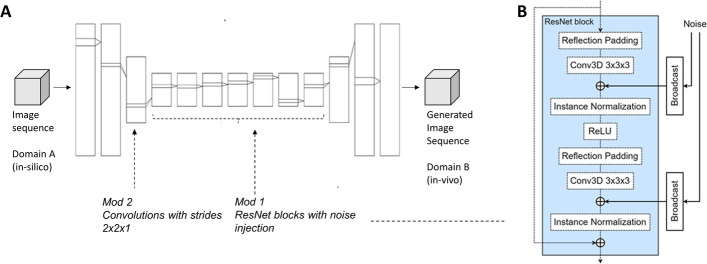
**A** Illustration of the cycleGAN generator architecture $G_{B}$, including two of the modifications made to the original model of cycleGAN [35]. **B** Illustration of the modification 1 made to the ResNet block in order to make the generator stochastic. Solid lines show the parts where modifications were made. The approach is similar to the StyleGAN model [45]

### Implementation

The implementation was based on the official implementation of CycleGAN^2^. The hyperparameters were set to default values unless stated otherwise. All convolutional layers, padding layers and instance normalization layers were replaced by their 3D counterparts. We used the ResNet6 block architecture for the generators and PatchGAN [38] for the discriminators. The GAN objective function was set to least-square GAN loss (LSGAN) that has proved to overcome vanishing gradients and loss saturation [51]. Fig. 4 illustrates the generator architecture of the cycleGAN and highlights some of the modifications that were implemented.

**Fig. 5:**
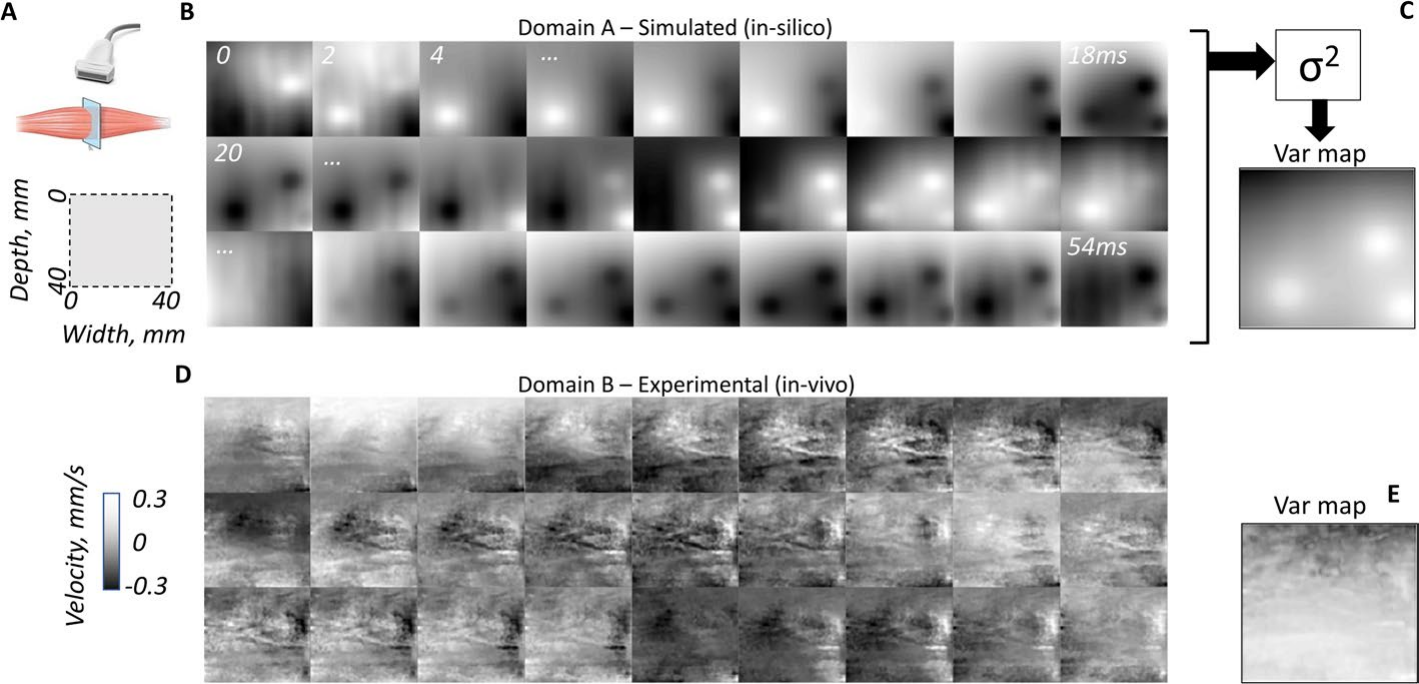
Examples of image sequences of skeletal intra-muscular contraction patterns from a cross-sectional image plane **A** of the biceps brachii muscle at a constant low force level. **B** Represents a simulated (in silico, domain A) image sequence, and in **C** its corresponding variance map (Var map), computed as the variance of the time signals at each pixel in the image-sequence. **D** Represents an experimental (in vivo, domain B) image sequence, and **E** its corresponding variation map. In both sequences, the oscillating behaviour of different spatial regions can be seen (putative contractions of motor units). The experimental images presented different spatial features as compared to the simulated ones. The time between consecutive frames is here 2 ms and images show colour-coded tissue velocity

### Modification 1—stochastic noise injection

The cycleGAN generators are deterministic and will have to fabricate experimental spatial features using pseudorandom number generators that are conditioned on the input. However, as described by [45], this consumes network capacity and hiding the intrinsic periodicity of the generated signal is difficult. Here, this problem was addressed as in the StyleGAN model [45] where per-pixel noise is added after every convolution. The added noise only affects stochastic features, leaving the overall composition and content intact [45].

In our implementation, the noise was added to the ResNet blocks and was broadcasted across the temporal dimension in an attempt to maintain the spatial features across all frames of the sequence (Fig. 4B). This modification makes the generator stochastic and it was only applied in the $G_{B}$ generator.

### Modification 2—stride in the temporal dimension

The 3D cycleGAN has a stride parameter which works in three dimensions—compared to a standard two-dimensional stride operator for 2D CNNs. Choosing any value higher than 1 at any layer will effectively down-sample the input to the layer. Hence, in the 3rd dimension of the 3D cycleGAN, which represents the temporal aspect of our data, we empirically chose a stride of 1 (default value is 2). This was implemented to ensure a maximal receptive field and temporal information of the muscle contractions.

### Modification 3—mixed precision format

The cycleGAN model is computationally and memory demanding. To reduce these issues, the number of features in the generator was halved similar to what [44] did. The models were also modified to run in mixed precision where most operations including convolutions operate on 16-bit floats instead of 32-bit floats which is significantly faster on modern GPUs and also reduces the memory consumption. Some layers like normalization layers require more precision and continue to operate on 32-bit floating points. Proper training with mixed precision achieves the same accuracy as single precision training [52].

### Modification 4—regularization of the generator

CycleGAN lacks supervision with a direct reconstruction error between $G_{B}(A)$ and *B* or $G_{A}(B)$ and *A*, which brings some uncertainty and difficulties towards the desired outputs. When data are transformed from one domain to the other, it can become distorted [53]. The distortion can then be recovered when the data are transformed back to the original domain. If the distortion does not impact the synthesized data, the undesired behaviour will not be penalized by the discriminator and the bijective transformation that causes the distortion will not be noticed in the cycle-consistency loss causing the distortion to remain. This problem also occurred when transforming image sequences between the simulated and experimental domains as the generators invert the activation of the MUs. For example, when transforming a contracting MU from the simulated domain to the experimental domain it becomes relaxed and then, when transforming it back to the simulated domain, it becomes contracted again. Ideally the discriminator should be able to notice this behaviour since motor units spend more time in relaxed state than in contracted state but the discriminators might not have enough frames to detect this pattern. This problem was solved by regularizing the generators with the identity mapping [54]:

5 $$\begin{aligned} \begin{aligned} {\mathcal {L}}_{\text{ idt}_A}={\mathbb {E}}_{a \sim {p_\text{ data }}(A)} \Vert G_{A}(a) - a \Vert _1, \\ {\mathcal {L}}_{\text{ idt}_B}={\mathbb {E}}_{b \sim {p_\text{ data }}(B)} \Vert G_{B}(b) - b \Vert _2. \end{aligned} \end{aligned}$$

### Training parameters

Training was performed on two RTX 2080 Ti GPUs. With a batch size of 2, the training took around 24 hours. The model was allowed to train for 80 epochs with a constant learning rate of 0.0002. The optimal performance was received after 60 epochs. The training dataset contained 100 examples for each domain with each example consisting of 1024 frames (2 seconds). During training, these image sequences were split into 32 image sequences of 32 frames each. During inference, however, we can process as many images as fits into memory since the generators are 3 dimensional and fully convolutional. Image sequences were randomly selected during training and vertical flipping was applied as data augmentation.

The number of parameters in the different included models are described in detail in Table 2 of the Appendix. The number of parameters were similar in all proposed modifications of the 3D cycleGAN model.

### Evaluation of translation performance

Several models for translation were compared. First, three models with various number of frames: 2D Cycle GAN (1 frame), recycle GAN (3 frames) and 3D Cycle GAN (32 frames). Note that 1 frame corresponds to approximately 2 ms, and 32 frames correspond to 64 ms, which should be enough to capture the contraction phase of the content signals [3–5]. Next, different modifications to the 3D CycleGAN model were compared: Original 3D CycleGAN, modification to stride 1 in 3rd dimension of the convolutions, and noise injection. Features of the translated data were compared with those of experimental data to determine the performance. All sequences (N=64) of the translated data were cross-compared with all sequences (N=64) of another domain and the mean and standard deviation of the performance metrics were computed. Three categories of metrics were compared: 1) spatial features, 2) temporal features, and 3) translated content consistency. Note: In this work, we consider image sequences of skeletal muscle contractions at a constant force. During such contractions, the central nervous system maintains force by repeated electrical depolarizations of the muscle fibres at a typical rate of 8-20Hz, resulting in an oscillating mechanical motion (consecutive contraction-relaxations) on a micrometre-level [5] (see Fig. 5B and 2A, D, G).

*Spatial features* were assessed using variance maps of the image sequences (Figure 5C, E). The variance maps were log-transformed to suppress potential high amplitude peaks of MU activations while retaining the spatial texture. The spatial features of translated sequences and experimental sequences were compared using several metrics. First, the distributions were compared using Histogram correlation and Bhattacharyya distance [55]. In addition, the spatial frequency content was compared using the structural similarity index measure (SSIM) [56] of a variance map’s corresponding K-space (2D Fourier transformed space, $k_{x}>0$ and $k_{y}>0$). The SSIM computes a similarity metric based on luminescence, contrast and structure features and ranges from 0 to 1 (high value indicates high similarity).

*Temporal features* were compared using correlation between the log-transformed power spectral densities (Corr Psd) of translated and experimental sequences. The Psd of a sequence was computed as the average of the Psds of the time signals of all the pixel positions.

*Translation content consistency* was assessed using cross-correlation between the paired time signals of an input sequence (in silico) and its corresponding translated sequence, from each pixel position. The maximal correlation and corresponding lag was computed. For each compared combination of input–output sequences, the mean and standard deviation of the correlations and lags were computed.

### Assessment of the learned mapping of the generator

In order to assess the mapping that the generator $G_B$ had learned, we computed difference maps between the paired input (simulated) and output (translated) domains. The difference maps were calculated for each frame and then averaged over all frames of a sequence (Fig. 3B).

## Datasets

The image sequences used in this work represent tissue velocity image sequences (TVI) from a cross-sectional plane of skeletal muscle tissue (Fig. 5A) contracting in an isometric mode (constant force, stable pool of active MUs). Thus, they are not grayscale B-mode ultrasound images representing structures and anatomy, but rather provide information on the subtle mechanical dynamics during contraction. Assuming that the fibres of the muscle are aligned approximately parallel, a cross-sectional plane will provide a representation of all MUs within that field of view. [5]. Domain ${\mathbf {A}}$ and ${\mathbf {B}}$ datasets correspond to simulated (in silico) data and experimental (in vivo) data, respectively. All image sequences from both the experimental and simulated domains have the dimensions of 128$\times$128$\times$1024 (corresponding to a cross–sectional area of the muscle of 40$\times$40 mm$^2$ and 2 seconds). Prior to training we standardized the datasets to ${\mathcal {N}}(0,1)$.

### Simulation dataset—domain A

In silico data of a skeletal muscle contraction at low contraction level (1-5% of maximal voluntary contraction level, MVC), were simulated using a previously described model in a cross-sectional image plane of a muscle [29]. The model simulates a TVI sequence of a contracting muscle at a constant force level based on superposition of mechanical twitches from included MUs. The simulation parameters were set to mimic the low MVC isometric contractions of the experimental data. The number of active MUs were 4-10 (uniform distribution), the positions of MUs were randomly distributed (uniform distribution in whole cross–section), and firing rate (*FR*) was between 8 and 13Hz (uniform distribution). The inter-pulse-interval was ${\mathcal {N}}(1/FR,0.2 \times 1/FR)$, 10% of a given MU’s firings were synchronized with firings of other MUs, and MU territory was assumed to be circular with diameters between 2.5 to 10mm (uniform distribution). It should be stressed that this simulation model only generates information on the contraction of the MU’s muscle fibres, and no interaction with other tissues are included. 128 sequences for training and 64 sequences for testing were generated using the simulation model.

### Experimental dataset—domain B

The experimental domain data, consisting of 64 image sequences, were acquired in a previous study [5]. Ultrasound image sequences were recorded from nine healthy subjects (27-45 years old, four men and five women) at weak isometric force level lasting 2 seconds. High frame rate (2kHz) plane wave ultrasound imaging was carried out in a cross-sectional image plane at 40 mm depth of the biceps brachi using a SonixTouch system (Ultrasonix Medical Corporation, Richmond, CA), L14-5 probe and DAQ module. 128-channel radiofrequency-image sequences were reconstructed using beamforming. TVI sequences were subsequently computed using a 2-D autocorrelation approach [57]. All processing details can be found in Rohlén et al. [5]. The temporal signals for each pixel of a TVI sequence was then passed through a 4th-order Butterworth bandpass filter at 5 to 50Hz. All images of a sequence were then passed through a 2D median filter with a kernel size of 1x1mm. Finally, to match the dimension of the simulated data, the filtered TVI sequence was down-sampled using bicubic spline interpolation to the dimensions 128 px $\times$ 128 px $\times$ 1024 samples and a sample rate of 512Hz. From a total of 64 examples of experimental data, 56 were randomly selected for training purpose and the remaining 8 were used for testing purpose. The subjects gave written informed consent, and the project conformed to the Declaration of Helsinki and was approved by the Swedish Ethical Review Authority (dnr 2019-01843).

## Data Availability

Not applicable.

## Appendix

Table 2 shows the number of parameters for each model.

**Table 2:** Number of parameters of the included models

Model	Number of parameters
2D cycleGAN (default)	28256644
recycleGAN	111897734
3D cycleGAN (default)	17322436
3D cycleGAN + stride 1	17322436
3D cycleGAN + stride 1 + noise injection	17322448

## Abbreviations

cycleGAN: Cycle generative adversarial network
MUs: Motor units
GANs: Generative adversarial networks
fMRI: Functional magnetic resonance imaging
D Bhat: Bhattacharyya distance
Corr: Histogram correlation
SSI: Structural similarity index of K-spaces
Corr Psd: Correlation of powerspectral densities
xCorr: Cross-correlation
Lag: Corresponding time lag
LSGAN: Least-square GAN loss
SSIM: Structural similarity index measure
TVI: Tissue velocity image sequences
MVC: Maximal voluntary contraction
FR: Firing rate

## Footnotes

1 The terms *simulation domain, in silico* and *domain* **A** refer to the same domain and are used interchangeably in this paper. The terms *experimental domain, in vivo* and *domain* **B** refer to the same domain and are used interchangeably in this paper. Note. Sim: simulation domain. Exp: experimental domain

2 https://github.com/junyanz/pytorch-CycleGAN-and-pix2pix.
